# The DRD2 Taq1A polymorphism moderates the effect of PTSD symptom severity on the left hippocampal CA3 volume: a pilot study

**DOI:** 10.1007/s00213-021-05882-z

**Published:** 2021-06-04

**Authors:** Minlan Yuan, Hongru Zhu, Yuchen Li, Fenfen Ge, Su Lui, Qiyong Gong, Changjian Qiu, Huan Song, Wei Zhang

**Affiliations:** 1 Mental Health Center and Psychiatric Laboratory, the State Key Laboratory of Biotherapy, West China Hospital of Sichuan University, 610041 Chengdu, China; 2 Huaxi Brain Research Center, West China Hospital of Sichuan University, Chengdu, China; 3 Huaxi MR Research Center (HMRRC), Department of Radiology, West China Hospital of Sichuan University, Chengdu, Sichuan China; 4 Radiology Department of the Second Affiliated Hospital, Wenzhou Medical University, Wenzhou, Zhejiang China; 5 West China Biomedical Big Data Center, West China Hospital, Sichuan University, 610041 Chengdu, China; 6 Medical Big Data Center, Sichuan University, Chengdu, 610041 China

**Keywords:** Posttraumatic stress disorder, Cornu ammonis, Dopamine receptor D2, Single nucleotide polymorphisms

## Abstract

**Rationale and objectives:**

The hippocampus, especially the CA1, CA3, and dentate gyrus (DG) subfields, is reported to be associated with post-traumatic stress disorder (PTSD) after trauma. However, neuroimaging studies of the associations between PTSD and hippocampal subfield volumes have failed to yield consistent findings. The aim of this study is to examine whether the dopamine D2 receptor (DRD2) Taq1A polymorphism, which is associated with both hippocampal function and PTSD, moderated the association between PTSD severity and hippocampal CA1, CA3 and DG volumes.

**Methods:**

T1-weighted images were acquired from 142 trauma survivors from the 2008 Wenchuan earthquake using a 3.0-T magnetic resonance imaging system. Hippocampal subfield segmentations were performed with FreeSurfer v6.0. We used the simple moderation model from the PROCESS v3.4 tool for SPSS 23.0 to examine the association between the rs1800497 polymorphism, PTSD severity, and hippocampal CA3 and DG volumes.

**Results:**

A significant genotype × PTSD symptom severity interaction was found for the left CA3 volume (Δ*F* = 5.01, *p* = 0.008, Δ*R*^2^ = 0.05). Post hoc, exploratory analyses deconstructing the interaction revealed that severe PTSD symptomatology were associated with reduced left CA3 volume among TC heterozygotes (*t* =  − 2.86, *p* = 0.005).

**Conclusions:**

This study suggests that DRD2 Taq1A polymorphism moderates the association between PTSD symptomatology and left CA3 volume, which promotes an etiological understanding of the hippocampal atrophy at the subfield level. This highlights the complex effect of environmental stress, and provides possible mechanism for the relationship between the dopaminergic system and hippocampal function in PTSD.

## Introduction

Individuals exposed to severe psychological or physically life-threatening traumatic events may develop posttraumatic stress disorder (PTSD), a debilitating condition with higher rates of suicide (Ramsawh et al. 2014), substance abuse (Kilpatrick et al. 2003), and major somatic diseases (Song et al. 2019). The hippocampus is thought to be functionally important for the pathogenesis and maintenance of PTSD symptoms, given its extensive involvement in memory processing after trauma (Cursano et al. 2020; Wingenfeld and Wolf 2014). Numerous neuroimaging studies of PTSD have reported smaller hippocampal subfields to be associated with PTSD (Hayes et al. 2017; Postel et al. 2019), while other studies fail to link PTSD with subfield differences in the hippocampus (Mueller et al. 2015). The discrepancies of these studies may be explained by several moderating variables, including genetic factors.

Although the association between PTSD and a particular gene has not been revealed by genome-wide association studies (Duncan et al. 2018), several candidate-gene studies of PTSD have reported associations with genes mainly including the serotonergic system, the stress response system and the dopaminergic system (Guillen-Burgos and Gutierrez-Ruiz 2018). Given that the dopaminergic signaling system plays an important role in many neural processes, such as reward and motivation, memory and learning, and motor behavior (Girault and Greengard 2004), the dopamine genetic variation has received substantial interest from investigators studying neuropsychiatric conditions such as PTSD (Banerjee et al. 2017), attention-deficit hyperactivity disorder (ADHD) (Pan et al. 2015), and schizophrenia (Yao et al. 2015).

In humans, the dopamine receptor D2 (DRD2) gene is located on Chr 11q23.2, encodes the dopamine D2 receptor, and harbors several genetic variants that have previously been associated with variations in D2 receptor expression (Grandy et al. 1989). The DRD2/ANKK1-Taq1A polymorphism (rs1800497) was previously assigned to DRD2, but later was found to be located on the neighboring ANKK1 gene. This polymorphism has been repeatedly found to be associated with the regulation of dopamine synthesis and reduced D2 receptor expression in the brain (Neville et al. 2004), which is the pathophysiological basis for various symptoms of PTSD (Gerlicher et al. 2018). Notably, a recent meta-analysis reported that the rs1800497 polymorphism in DRD2 showed significant association with PTSD (Li et al. 2016). An association of DRD2 rs1800497 with PTSD symptom severity was identified with the T allele as the risk allele (Hoxha et al. 2019).

DRD2 is a G-protein coupled receptor, expressed predominantly in the hippocampus (Gangarossa et al. 2012; Wei et al. 2018). Although the role of DRD2 in the hippocampus has been largely underexplored to date, a number of animal studies have proposed a role for D2 receptors in hippocampus-dependent memory acquisition and/or consolidation and depression expression (de Lima et al. 2011; Rocchetti et al. 2015). To date, whether individuals with a risk variant in the dopamine D2 receptor gene are more likely to suffer hippocampal subfield volume alterations in response to posttraumatic events remains unexplored.

Given the central role that hippocampal deficits play in PTSD (Woon et al. 2010), and the aforementioned association between the DRD2 Taq1A polymorphism and PTSD symptoms, the aim of the present study was to explore whether the DRD2 Taq1A polymorphism moderated the association between PTSD symptoms and hippocampal volume at the subfield level. Investigations on the impact of stress on the hippocampus at the subfield level show that stress in rats produces atrophy of pyramidal neurons and dendritic debranching in cornu ammonis (McEwen 2002) and suppression in neurogenesis in dentate gyrus (DG) (Mirescu and Gould 2006). In humans, hippocampal subfield volume alterations in CA1 (Chen et al. 2018), CA3 (Postel et al. 2019), and DG (Hayes et al. 2017) are mostly reported in PTSD across studies; therefore, we examined the moderating effect of Taq1A polymorphisms on the association between PTSD symptoms and bilateral CA1, CA3, and DG volumes. We hypothesized that the traumatized individuals with severe PTSD symptoms who are carriers of the DRD2 rs1800497 T allele were more likely to show hippocampal subfield volume reductions than C carriers.

## Methods

### Participants

We recruited earthquake survivors from the 2008 Wenchuan earthquake, which had a Richter Scale magnitude of 8.0 (Stone 2009), in the years 2012–2013 and 2015–2017. Participants were included if they were 18 to 60 years old, right-handed, had experienced or witnessed the trauma in an extreme disaster area, and had not received any psychiatric medication or regular psychotherapy. The exclusion criteria were as follows: (1) any history of neurological disease; (2) any history of other major psychiatric disorders such as schizophrenia, bipolar disorder, or alcohol and/or other substance abuse/dependence (comorbid depression and anxiety disorders were not excluded); (3) mental retardation; (4) major head injury involving loss of consciousness for more than 10 min; (5) contraindication to MRI imaging such as metal implants; (6) pregnancy. A total of 142 participants were included in the present study.

We obtained approval for the study from the Medical Ethics Committee of West China Hospital, Sichuan University, and written informed consent was obtained from all participants.

### Clinical assessments

Each participant was assessed using the Structured Clinical Interview for DSM-IV (SCID) (First et al. 1997) and the Clinical Administered PTSD Scale (CAPS) (Blake et al. 1995) by two psychiatrists with relevant training. The DSM-IV criteria were used because no formal Chinese version of the Structured Clinical Interview for DSM-V was available at the time of participant enrolment. The participants completed the life events checklist (Gray et al. 2004) to evaluate antecedent traumatization. Every participant confirmed that earthquake trauma was the most severe trauma in their lifetime; therefore, the index trauma for evaluating PTSD symptom severity was the earthquake. On the CAPS interview, a total score combining the frequency and intensity of the PTSD symptoms for all the DSM-IV PTSD criteria was derived. According to the DSM-IV, 69 of the 142 participants met with the diagnostic criteria of PTSD. The presence of lifetime depression and anxiety disorders assessed using the SCID included depressive disorder *N* = 32, agoraphobia *N* = 3, generalized anxiety disorder *N* = 1, and panic disorder *N* = 2. Five participants had both depressive and anxiety diagnoses; therefore, the number of participants with a diagnosis of depressive/anxiety disorder was 33.

To evaluate cognitive performance, participants were also assessed using the Wechsler Memory Scale-IV (WMS-IV) (Wechsler 2009). The results of these tests yielded five index scores: auditory memory index, visual memory index, visual working memory index, immediate memory index, and delayed memory index (Weiss et al. 2010).

### MRI acquisition and data processing

Participants were scanned on a 3.0-T MRI imaging system (Siemens 3.0 T Trio, Erlangen, Germany) with a 12-channel phased-array head coil, as described in our previous study (Yuan et al. 2019). T1-weighted images were acquired (slice thickness = 1 mm, TR/TE = 1900/2.26 ms, flip angle = 9°, FOV = 240 × 240 mm, number of slices = 176, data matrix = 256 × 256).

**Fig. 1:**
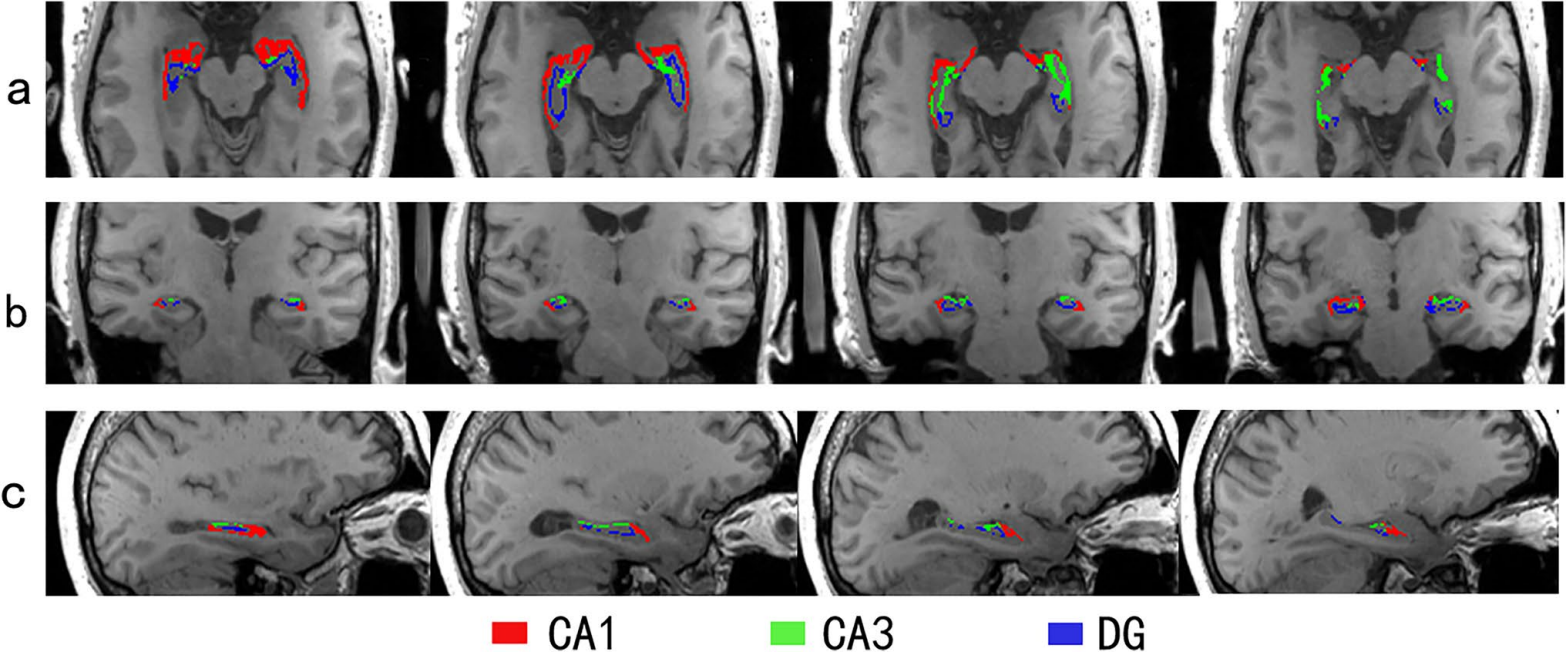
The bilateral CA1, CA3, and dentate gyrus (DG) in a participant's hippocampus. **a** Axial slices from the inferior to superior hippocampus, **b** coronal slices from the posterior to anterior hippocampus, and **c** sagittal slices from the lateral to medial hippocampus

Automated hippocampal subfield segmentations were performed with FreeSurfer v6.0, which is available for download online (http://surfer.nmr.mgh.harvard.edu/). The segmentation algorithm for hippocampal subfields was based on an atlas derived from high-resolution (0.13 mm) ex vivo MRI data of postmortem medial temporal tissue from a 7-T scanner, which was able to reliably identify the molecular layer of the DG, the CA regions, and the subiculum (Iglesias et al. 2015). Based on our literature review, we focused on three hippocampal subfields, CA1, CA3, and DG, which were reported to be associated with PTSD, as we discussed in the introduction (Chen et al. 2018; Hayes et al. 2017; McEwen 2002; Mirescu and Gould 2006; Postel et al. 2019). Figure 1 shows the automated hippocampal subfields of interest in a participant’s hippocampus. The estimated total intracranial volume (eTIV) of each participant was also calculated by the segmentation algorithm. To ensure segmentation accuracy and valid assignment of the hippocampal subfields, each segmented image was overlaid on the corresponding brain structural image and manually inspected by an investigator who was blind to the symptom severity of the participants.

### Genotyping

Peripheral blood samples were collected from each participant for genotyping, except two participants who refused to draw blood. Genomic DNA was isolated from peripheral white blood cells using the Wizard Genomic DNA Purification Kit (Promega, Madison, Wisconsin, USA). The DNA concentration of three samples was less than 20 ng/μL following the initial concentration determination by UV spectrophotometry, so they were excluded. Therefore, a total of 137 blood samples were finally genotyped. Genotyping of DRD2 Taq1A (rs1800497) was conducted using MALDI-TOF analysis on the Sequenom MassARRAY iPLEX platform (Gabriel et al. 2009). The call rate was 100% and the minor allele frequency was 40.62%, indicating good quality DNA and results. The genotype distribution in the all the participants did not deviate from Hardy–Weinberg equilibrium (*χ*^2^ = 0.02, *p* = 0.89).

### Statistical analyses

Statistical analyses were performed using SPSS software version 23 (IBM, Armonk, NY). The distribution of each continuous variable was examined for outliers or inconsistency in the total sample. The hippocampal subfield volumes and the CAPS score were not normally distributed. Demographic characteristics among genotype groups were compared using one-way ANOVA in the case of quantitative data. Pearson’s *χ*^2^ tests were used in case of qualitative data (i.e., gender and depression/anxiety diagnosis). The hippocampal volumes were log transformed to make them conform to a normal distribution. We used the simple moderation model from the PROCESS tool (Hayes 2017) for SPSS to examine the moderation effect of the genotype on the association between PTSD symptom severity and CA1, CA3, and DG volume. The volume of each hippocampal subfield was used as the dependent variable, with the CAPS score as the independent variable and age, gender, years of education, depression/anxiety diagnosis, and eTIV as covariates. The genotype of DRD2 rs1800497 was used as a multicategorical variable as the moderator. These models were run separately for the volumes of left CA1, right CA1, left CA3, right CA3, left DG, and right DG. Multiple comparisons were conducted using false discovery rate (FDR) corrections. Post hoc, exploratory analyses were performed on significant interactions by stratifying the sample by genotype, to deconstruct the effects of PTSD symptoms on hippocampal subfield volumes.

As previous studies mainly reported deficits in the whole hippocampal volume in traumatized subjects and PTSD patients (Woon et al. 2010), we also performed an exploratory analysis using the whole hippocampal volumes (left and right hemisphere separately) as independent variables, to investigate whether the DRD2 Taq1A polymorphism could moderate the effect of PTSD symptoms on the hippocampus as a whole relative to subfields.

## Results

### Descriptive statistics of genetic data and demographics

Genotyping yielded three groups: 50 (36.5%) trauma survivors carrying two C alleles (CC), 65 (47.4%) carrying one C and one T allele (TC), and 22 (16.1%) carrying two T alleles (TT). The groups did not differ significantly in age, gender, years of education, CAPS score, depression/anxiety diagnosis, or WMS-IV. The demographic characteristics of the sample are shown in Table 1.

**Table 1:** Demographic characteristics among genotypes

Characteristics	*N* = 142	CC (*n* = 50)	TC (*n* = 65)	TT (*n* = 22)	*F* or *χ*^2^	*p* value
Age	44 ± 11	42 ± 13	45 ± 9	43 ± 11	0.98	0.38
Gender (female/male)	95/47	34/16	44/21	14/8	0.15	0.93
Education	10 ± 9	11 ± 12	9 ± 6	10 ± 7	0.74	0.48
Left_CA1	643.95 ± 75.83	644.05 ± 85.15	644.97 ± 74.12	65.13 ± 13.89	0.006	0.99
Left_CA3	212.48 ± 29.95	214.02 ± 29.96	213.38 ± 32.12	206.68 ± 24.96	0.50	0.61
Left_DG	313.60 ± 34.11	314.99 ± 34.32	313.87 ± 36.26	311.77 ± 29.71	0.66	0.94
Right_CA1	666.23 ± 76.78	666.61 ± 78.16	666.76 ± 73.27	666.53 ± 85.22	< 0.001	1.00
Right_CA3	233.09 ± 32.74	236.51 ± 33.14	232.98 ± 34.04	224.20 ± 28.57	1.07	0.35
Right_DG	325.82 ± 36.02	327.75 ± 34.08	326.13 ± 38.91	319.14 ± 31.14	0.45	0.64
CAPS_total	40 ± 35	38 ± 34	40 ± 35	40 ± 36	0.53	0.95
WMS-IV^a^	92 ± 12	92 ± 13	93 ± 11	92 ± 12	0.21	0.81
Depression/anxiety diagnosis	33 (23.2%)	10 (20%)	16 (24.6%)	5 (22.7%)	0.34	0.84

Abbreviations: *DG*, dentate gyrus; *CAPS*, Clinical Administered PTSD Scale; *WMS-IV*, Wechsler Memory Scale-IV

^a^A total of 103 participants were assessed with the Wechsler Memory Scale-IV

### Moderating effect of DRD2 Taq1A polymorphism

**Fig. 2:**
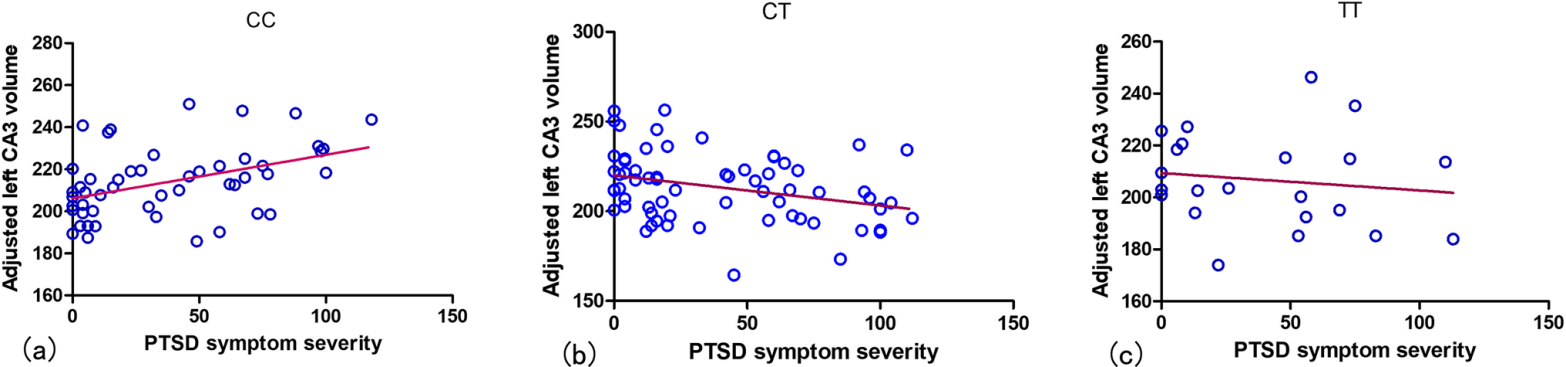
Partial regression plots accounting for covariates. **a** Individuals with the CC genotype showed increased left CA3 volume with increasing traumatic stress. **b** Individuals with the TC genotype showed reduced left CA3 volume with increasing traumatic stress. **c** These patterns were not observed in the TT genotype. PTSD, posttraumatic stress disorder

The simple moderation model in the PROCESS showed that after entering the genotype × CAPS score interaction in the model, there was a significant change in *F* (Δ*F* = 5.01, *p* = 0.008, Δ*R*^2^ = 0.05) for the left CA3 volume, withstanding FDR correction for multiple tests (*p* = 0.048), suggesting that the DRD2 Taq1A polymorphism moderated the effect of PTSD symptom on the volume of the left CA3. However, the polymorphism did not moderate the effect of PTSD symptoms on the volume of the right CA3, left CA1, right CA1, left DG, or right DG (Table 2). Post hoc, exploratory examination of the interaction term revealed that the CAPS score was positively associated with the left CA3 volume for the CC genotype (*β* = 0.30, *t* = 2.26, *p* = 0.029, Fig. 2a). In addition, individuals with the TC genotype showed reduced left hippocampal volume with increasing PTSD symptom severity (*β* =  − 0.28, *t* =  − 2.15, *p* = 0.036, Fig. 2b). There was no association between PTSD symptom severity and hippocampal volume in the TT group (*β* =  − 0.20, *t* =  − 1.51, *p* = 0.152, Fig. 2c). Exploratory analysis of the whole hippocampus showed that the polymorphism did not moderate the effect of PTSD symptoms on the volume of the entire left (Δ*F* = 0.47, *p* = 0.624, Δ*R*^2^ = 0.004) or right (Δ*F* = 0.24, *p* = 0.786, Δ*R*^2^ = 0.002) hippocampus, suggesting that subfield volumes are more sensitive to DRD2 Taq1A polymorphism moderation when exposed to stress.

**Table 2:** CAPS score × genotype interaction of the simple moderation models for the interested hippocampal subfields

Hippocampal subfields	Δ*R*^2^	Δ*F*_(2, 126)_	*p* value (uncorrected)	*p* value (corrected)
Left CA1	0.008	0.89	0.413	0.502
Right CA1	0.009	0.88	0.419	0.502
Left CA3	0.05	5.01	0.008	**0.048**
Right CA3	0.03	2.30	0.104	0.312
Left DG	0.01	1.25	0.289	0.385
Right DG	0.007	0.76	0.469	0.469

Multiple comparison correction with false discovery rate (FDR) for different hippocampal subfields. Bold value indicates statistical significance defined as *p* < 0.05

Abbreviations: *DG*, dentate gyrus; *CAPS*, Clinical Administered PTSD Scale

## Discussion

To our knowledge, this is the first study exploring DRD2 polymorphism interactions with PTSD symptom severity in predicting hippocampal subfield volume in a sample of traumatized individuals. A significant moderating effect of the DRD2 Taq1A polymorphism on the association between PTSD symptoms and left CA3 volume was found. Specifically, severe PTSD symptomatology was associated with reduced left CA3 volume among TC heterozygotes, while in CC homozygotes, greater current PTSD symptom severity showed a significant increase in left CA3 volume. A genetic moderation effect on the association between PTSD symptoms and the whole hippocampal volume was not observed. These findings indicated that the association between PTSD and hippocampal volume in previous studies might be confounded by genotype-dependent exposure differences, as no hippocampal atrophy could be detected for individuals who lacked a genetic vulnerability.

The smaller hippocampus in PTSD has been traditionally attributed to stress-induced release of glucocorticoids, which can alter hippocampal microarchitecture and cell number (Neylan et al. 2003; Sapolsky 2000; Zhao et al. 2007). However, studies have suggested a relationship between dopamine activity and synaptic long-term potentiation (LTP) in the hippocampus (Li et al. 2003; Rocchetti et al. 2015). LTP is reported to be associated with neurogenesis in the hippocampus, both in terms of the proliferation of progenitor cells and the survival of new cells (Bruel-Jungerman et al. 2006). Furthermore, the CA3 hippocampus has been one of the prototypical sites of LTP in animal studies (Debanne et al. 1996; Do et al. 2002; Minami et al. 2016; Shin et al. 2016). Evidence has shown that stress inhibits dopamine-modulated LTP (Jay et al. 2004; Rocher et al. 2004), taken together, these findings may explain the interaction between traumatic stress symptoms and dopamine-mediated processes on the hippocampal subfield CA3. We found the moderation effect of the DRD2 Taq1A genetic polymorphism unilaterally on the left CA3, which was in line with previous studies (O’Doherty et al. 2015). However, further investigations are needed to examine how dopamine activity moderates the association between stress and the left CA3 and other hippocampal subfields.

The DRD2 Taq1A polymorphism has consistently been found to be associated with the regulation of dopamine synthesis and reduced D2 receptor expression in the brain (Neville et al. 2004); specifically, the presence of T allele carriers compared to CC homozygotes is associated with reduced receptor density (Montag et al. 2010; Pohjalainen et al. 1998). The result that TC genotype carriers were at risk for reduced hippocampal subfield volume is in agreement with other studies showing that T allele carriers of the DRD2 polymorphism had worse performance on cognitive tasks (Berryhill et al. 2013; Jocham et al. 2009; Persson et al. 2015). Animal studies indicate that astrocytic DRD2 activation normally suppresses neuroinflammation in the central nervous system through an αB-crystallin-dependent mechanism (Shao et al. 2013). Therefore, lower D2 receptor density in T allele carriers in the present study may be associated with higher levels of neuroinflammation, which is likely to cause hippocampal atrophy. On the other hand, a larger left CA3 volume in DRD2 Taq1 T non-carriers, as was shown in CC genotype carriers in our study, presumably has higher D2 receptor density and, by extension, lower levels of neuroinflammation, which may support lager gray matter volume. Speculatively, the phenomenon that some individuals present good cognitive performance even with the same degree of PTSD symptom severity (Haglund et al. 2007) may be influenced by C homozygosity as a protective factor.

Several limitations should be noted for the present study, the first of which was the small sample size (*n* = 142). This may explain the post hoc results showing that the negative association between PTSD symptom severity and the left CA3 volume was found only in the TC group (*n* = 65) but not in the TT group (*n* = 22). Studies with a larger sample are needed to confirm our findings. Second, only one SNP of the DRD2 gene was examined to explore a moderation effect between PTSD and hippocampal subfield volume due to financial constraints. Although the Taq1A SNP (rs1800497) is the most commonly studied genetic variation in different psychiatric disorders, more variations in the DRD2 gene should be examined to obtain a comprehensive assessment of genetic information. Therefore, the present findings were preliminary and need to be replicated in another traumatized sample. Third, because about 73 out of 142 participants did not met diagnostic criteria for PTSD, the PTSD symptoms of these participants may be mild. The variability (or lack thereof) of PTSD symptoms could introduce overestimation or underestimation effects on the outcomes. Therefore, a larger traumatized sample is needed to further validate our findings. Fourth, our hypotheses about the influence of dopamine-moderated hippocampal subfield volume reduction on memory dysfunction should be further verified; nevertheless, these findings provide implications for future studies integrating genotyping of dopaminergic system risk loci and recently developed imaging techniques to help the detection of PTSD.

## Conclusion

In conclusion, despite the aforementioned limitations, this study is the first to provide preliminary evidence that the T allele carriers of DRD2 Taq1A are more likely to show reductions in hippocampal subfield volume with severe PTSD symptoms, which implies possible direction for molecular functional studies in the future. These findings are notable, as previous studies of the relationship between traumatic stress and hippocampal volume have produced inconsistent results, suggesting the need to examine the genetic variation to improve specificity. The left CA3 may be a hippocampal subfield vulnerable to dopamine-mediated process under traumatic stress. This study supports and extends the findings of previous studies by promoting an etiological understanding of the hippocampal atrophy at the subfield level. This highlights the complex effect of environmental stress, and provides possible mechanism for the relationship between the dopaminergic system and hippocampal function in PTSD.

## References

[CR1] Banerjee SB, Morrison FG, Ressler KJ (2017). Genetic approaches for the study of PTSD: advances and challenges. Neurosci Lett.

[CR2] Berryhill ME, Wiener M, Stephens JA, Lohoff FW, Coslett HB, Zhang H (2013). COMT and ANKK1-Taq-Ia Genetic Polymorphisms Influence Visual Working Memory. PLoS ONE.

[CR3] Blake DD, Weathers FW, Nagy LM, Kaloupek DG, Gusman FD, Charney DS, Keane TM (1995). The development of a Clinician-Administered PTSD Scale. J Trauma Stress.

[CR4] Bruel-Jungerman E, Davis S, Rampon C, Laroche S (2006). Long-term potentiation enhances neurogenesis in the adult dentate gyrus. J Neurosci.

[CR5] Chen LW, Sun D, Davis SL, Haswell CC, Dennis EL, Swanson CA, Whelan CD, Gutman B, Jahanshad N, Iglesias JE, Thompson P, Mid-Atlantic MW, Wagner HR, Saemann P, LaBar KS, Morey RA (2018). Smaller hippocampal CA1 subfield volume in posttraumatic stress disorder. Depress Anxiety.

[CR6] Cursano S, Battaglia CR, Urrutia-Ruiz C, Grabrucker S, Schon M, Bockmann J, Braumuller S, Radermacher P, Roselli F, Huber-Lang M, Boeckers TM (2020) A CRHR1 antagonist prevents synaptic loss and memory deficits in a trauma-induced delirium-like syndrome. Mol Psychiatry. 10.1038/s41380-020-0659-y PMC8550963 32051550

[CR7] de Lima MNM, Presti-Torres J, Dornelles A, Scalco FS, Roesler R, Garcia VA, Schröder N (2011). Modulatory influence of dopamine receptors on consolidation of object recognition memory. Neurobiol Learn Mem.

[CR8] Debanne D, Gähwiler BH, Thompson SM (1996). Cooperative interactions in the induction of long-term potentiation and depression of synaptic excitation between hippocampal CA3–CA1 cell pairs in vitro. Proc Natl Acad Sci.

[CR9] Do VH, Martinez CO, Martinez JL, Derrick BE (2002). Long-term potentiation in direct perforant path projections to the hippocampal CA3 region in vivo. J Neurophysiol.

[CR10] Duncan LE, Ratanatharathorn A, Aiello AE, Almli LM, Amstadter AB, Ashley-Koch AE, Baker DG, Beckham JC, Bierut LJ, Bisson J (2018). Largest GWAS of PTSD (N = 20 070) yields genetic overlap with schizophrenia and sex differences in heritability. Mol Psychiatry.

[CR11] First M, Spitzer RL, Gibbon M (1997). Structured clinical interview for DSM-IV axis I disorders.

[CR12] Gabriel S, Ziaugra L, Tabbaa D (2009) SNP genotyping using the Sequenom MassARRAY iPLEX platform. Current protocols in human genetics 60: 2.12. 1–2.12. 18. 10.1002/0471142905.hg0212s60 19170031

[CR13] Gangarossa G, Longueville S, De Bundel D, Perroy J, Hervé D, Girault JA, Valjent E (2012). Characterization of dopamine D1 and D2 receptor-expressing neurons in the mouse hippocampus. Hippocampus.

[CR14] Gerlicher A, Tüscher O, Kalisch R (2018). Dopamine-dependent prefrontal reactivations explain long-term benefit of fear extinction. Nat Commun.

[CR15] Girault JA, Greengard P (2004). The neurobiology of dopamine signaling. Arch Neurol.

[CR16] Grandy DK, Litt M, Allen L, Bunzow JR, Marchionni M, Makam H, Reed L, Magenis RE, Civelli O (1989). The human dopamine D2 receptor gene is located on chromosome 11 at q22–q23 and identifies a TaqI RFLP. Am J Hum Genet.

[CR17] Gray MJ, Litz BT, Hsu JL, Lombardo TW (2004). Psychometric properties of the life events checklist. Assessment.

[CR18] Guillen-Burgos HF, Gutierrez-Ruiz K (2018). Genetic advances in post-traumatic stress disorder. Rev Colomb Psiquiatr.

[CR19] Haglund M, Cooper N, Southwick S, Charney D (2007). keys to resilience for PTSD and everyday stress. Current Psychiatry.

[CR20] Hayes AF (2017) Introduction to mediation, moderation, and conditional process analysis: a regression-based approach. Guilford publications

[CR21] Hayes JP, Hayes S, Miller DR, Lafleche G, Logue MW, Verfaellie M (2017). Automated measurement of hippocampal subfields in PTSD: evidence for smaller dentate gyrus volume. J Psychiatr Res.

[CR22] Hoxha B, Goci Uka A, Agani F, Haxhibeqiri S, Haxhibeqiri V, Sabic Dzananovic E, Kucukalic S, Bravo Mehmedbasic A, Kucukalic A, Dzubur Kulenovic A, Feric Bojic E, Marjanovic D, Kravic N, Avdibegovic E, Muminovic Umihanic M, Jaksic N, Cima Franc A, Rudan D, Jakovljevic M, Babic R, Pavlovic M, Babic D, Aukst Margetic B, Bozina N, Sinanovic O, Ziegler C, Warrings B, Domschke K, Deckert J, Wolf C, Vyshka G (2019). The role of TaqI DRD2 (rs1800497) and DRD4 VNTR polymorphisms in posttraumatic stress disorder (PTSD). Psychiatr Danub.

[CR23] Iglesias JE, Augustinack JC, Nguyen K, Player CM, Player A, Wright M, Roy N, Frosch MP, McKee AC, Wald LL, Fischl B, Van Leemput K (2015). A computational atlas of the hippocampal formation using ex vivo, ultra-high resolution MRI: application to adaptive segmentation of in vivo MRI. NeuroImage.

[CR24] Jay TM, Rocher C, Hotte M, Naudon L, Gurden H, Spedding M (2004). Plasticity at hippocampal to prefrontal cortex synapses is impaired by loss of dopamine and stress: importance for psychiatric diseases. Neurotox Res.

[CR25] Jocham G., Klein T. A., Neumann J., von Cramon D. Y., Reuter M., Ullsperger M. (2009). Dopamine DRD2 Polymorphism Alters Reversal Learning and Associated Neural Activity. Journal of Neuroscience.

[CR26] Kilpatrick DG, Ruggiero KJ, Acierno R, Saunders BE, Resnick HS, Best CL (2003). Violence and risk of PTSD, major depression, substance abuse/dependence, and comorbidity: results from the National Survey of Adolescents. J Consult Clin Psychol.

[CR27] Li L, Bao Y, He S, Wang G, Guan Y, Ma D, Wang P, Huang X, Tao S, Zhang D, Liu Q, Wang Y, Yang J (2016). The association between genetic variants in the dopaminergic system and posttraumatic stress disorder: a meta-analysis. Medicine (baltimore).

[CR28] Li S, Cullen WK, Anwyl R, Rowan MJ (2003). Dopamine-dependent facilitation of LTP induction in hippocampal CA1 by exposure to spatial novelty. Nat Neurosci.

[CR29] McEwen BS (2002). Sex, stress and the hippocampus: allostasis, allostatic load and the aging process. Neurobiol Aging.

[CR30] Minami A, Saito M, Mamada S, Ieno D, Hikita T, Takahashi T, Otsubo T, Ikeda K, Suzuki T (2016). Role of sialidase in long-term potentiation at mossy fiber-CA3 synapses and hippocampus-dependent spatial memory. PLoS ONE.

[CR31] Mirescu C, Gould E (2006). Stress and adult neurogenesis. Hippocampus.

[CR32] Montag C, Markett S, Basten U, Stelzel C, Fiebach C, Canli T, Reuter M (2010). Epistasis of the DRD2/ANKK1 Taq Ia and the BDNF Val66Met polymorphism impacts novelty seeking and harm avoidance. Neuropsychopharmacology.

[CR33] Mueller SG, Ng P, Neylan T, Mackin S, Wolkowitz O, Mellon S, Yan X, Flory J, Yehuda R, Marmar CR (2015). Evidence for disrupted gray matter structural connectivity in posttraumatic stress disorder. Psychiatry Res.

[CR34] Neville MJ, Johnstone EC, Walton RT (2004). Identification and characterization of ANKK1: a novel kinase gene closely linked to DRD2 on chromosome band 11q23.1. Hum Mutat.

[CR35] Neylan TC, Schuff N, Lenoci M, Yehuda R, Weiner MW, Marmar CR (2003). Cortisol levels are positively correlated with hippocampal N-acetylaspartate. Biol Psychiatry.

[CR36] O'Doherty DC, Chitty KM, Saddiqui S, Bennett MR, Lagopoulos J (2015). A systematic review and meta-analysis of magnetic resonance imaging measurement of structural volumes in posttraumatic stress disorder. Psychiatry Res.

[CR37] Pan Y-Q, Qiao L, Xue X-D, Fu J-H (2015). Association between ANKK1 (rs1800497) polymorphism of DRD2 gene and attention deficit hyperactivity disorder: a meta-analysis. Neurosci Lett.

[CR38] Persson Jonas, Rieckmann Anna, Kalpouzos Grégoria, Fischer Håkan, Bäckman Lars (2015). Influences of a DRD2 polymorphism on updating of long-term memory representations and caudate BOLD activity: magnification in aging. Human Brain Mapping.

[CR39] Pohjalainen T, Rinne JO, Någren K, Lehikoinen P, Anttila K, Syvälahti EK, Hietala J (1998). The A1 allele of the human D2 dopamine receptor gene predicts low D2 receptor availability in healthy volunteers. Mol Psychiatry.

[CR40] Postel C, Viard A, André C, Guénolé F, de Flores R, Baleyte JM, Gerardin P, Eustache F, Dayan J, Guillery-Girard B (2019). Hippocampal subfields alterations in adolescents with post-traumatic stress disorder. Hum Brain Mapp.

[CR41] Ramsawh HJ, Fullerton CS, Mash HBH, Ng THH, Kessler RC, Stein MB, Ursano RJ (2014). Risk for suicidal behaviors associated with PTSD, depression, and their comorbidity in the US Army. J Affect Disord.

[CR42] Rocchetti J, Isingrini E, Dal Bo G, Sagheby S, Menegaux A, Tronche F, Levesque D, Moquin L, Gratton A, Wong TP (2015). Presynaptic D2 dopamine receptors control long-term depression expression and memory processes in the temporal hippocampus. Biol Psychiat.

[CR43] Rocher C, Spedding M, Munoz C, Jay TM (2004). Acute stress-induced changes in hippocampal/prefrontal circuits in rats: effects of antidepressants. Cereb Cortex.

[CR44] Sapolsky RM (2000). Glucocorticoids and hippocampal atrophy in neuropsychiatric disorders. Arch Gen Psychiatry.

[CR45] Shao W, Zhang S-z, Tang M, Zhang X-h, Zhou Z, Yin Y-q, Zhou Q-b, Huang Y-y, Liu Y-j, Wawrousek E (2013). Suppression of neuroinflammation by astrocytic dopamine D2 receptors via αB-crystallin. Nature.

[CR46] Shin S, Han S, Woo R-S, Jang S, Min S (2016). Adolescent mice show anxiety-and aggressive-like behavior and the reduction of long-term potentiation in mossy fiber-CA3 synapses after neonatal maternal separation. Neuroscience.

[CR47] Song H, Fang F, Arnberg FK, Mataix-Cols D, de la Cruz LF, Almqvist C, Fall K, Lichtenstein P, Thorgeirsson G, Valdimarsdóttir UA (2019) Stress related disorders and risk of cardiovascular disease: population based, sibling controlled cohort study. bmj 365: l1255. 10.1136/bmj.l1255 PMC6457109 30971390

[CR48] Stone R (2009) A deeply scarred land. American Association for the Advancement of Science

[CR49] Wechsler D (2009). Wechsler memory scale–fourth edition (WMS-IV).

[CR50] Wei X, Ma T, Cheng Y, Huang CCY, Wang X, Lu J, Wang J (2018). Dopamine D1 or D2 receptor-expressing neurons in the central nervous system. Addict Biol.

[CR51] Weiss LG, Saklofske DH, Coalson D, Raiford SE (2010). WAIS-IV clinical use and interpretation: scientist-practitioner perspectives.

[CR52] Wingenfeld K, Wolf OT (2014). Stress, memory, and the hippocampus. Hippocampus Clin Neurosci.

[CR53] Woon FL, Sood S, Hedges DW (2010). Hippocampal volume deficits associated with exposure to psychological trauma and posttraumatic stress disorder in adults: a meta-analysis. Prog Neuropsychopharmacol Biol Psychiatry.

[CR54] Yao J, Yq P, Ding M, Pang H, Bj W (2015). Association between DRD2 (rs1799732 and rs1801028) and ANKK1 (rs1800497) polymorphisms and schizophrenia: a meta-analysis. Am J Med Genet B Neuropsychiatr Genet.

[CR55] Yuan M, Pantazatos SP, Zhu H, Li Y, Miller JM, Rubin-Falcone H, Zanderigo F, Ren Z, Yuan C, Lui S, Gong Q, Qiu C, Zhang W, John Mann J (2019). Altered amygdala subregion-related circuits in treatment-naive post-traumatic stress disorder comorbid with major depressive disorder. Eur Neuropsychopharmacol.

[CR56] Zhao H, Xu H, Xu X, Young D (2007). Predatory stress induces hippocampal cell death by apoptosis in rats. Neurosci Lett.

